# The Window of Desiccation Tolerance Shown by Early-Stage Germinating Seedlings Remains Open in the Resurrection Plant, *Xerophyta viscosa*

**DOI:** 10.1371/journal.pone.0093093

**Published:** 2014-03-25

**Authors:** Rafe Lyall, Robert A. Ingle, Nicola Illing

**Affiliations:** Department of Molecular and Cell Biology, University of Cape Town, Rondebosch, South Africa; University of North Carolina at Chapel Hill, United States of America

## Abstract

Resurrection plants are renowned for their vegetative desiccation tolerance (DT). While DT in vegetative tissues is rare in angiosperms, it is ubiquitous in mature orthodox seeds. During germination, seedlings gradually lose DT until they pass a point of no return, after which they can no longer survive dehydration. Here we investigate whether seedlings of the resurrection plant *Xerophyta viscosa* ever lose the capacity to establish DT. Seedlings from different stages of germination were dehydrated for 48 hours and assessed for their ability to recover upon rehydration. While a transient decline in the ability of *X. viscosa* seedlings to survive dehydration was observed, at no point during germination was the ability to re-establish DT completely lost in all seedlings. Pre-treatment of seedlings with PEG or sucrose reduced this transient decline, and improved the survival rate at all stages of germination. Additionally, we observed that the trait of poikilochlorophylly (or loss of chlorophyll) observed in adult *X. viscosa* leaves can be induced throughout seedling development. These results suggest that the window of DT seen in germinating orthodox seeds remains open in *X. viscosa* seedlings and that vegetative DT in *Xerophyta* species may have evolved from the ability to retain this program through to adulthood.

## Introduction

Desiccation tolerance (DT) is the ability of an organism to revive unharmed after almost complete loss of cellular water from its tissues [1]. While vegetative DT is relatively common in mosses and liverworts, it is extremely rare in angiosperms, having been reported from only 135 taxonomically diverse species from 44 genera collectively known as resurrection plants [2]. Though rare in vegetative tissue, DT is common in the reproductive tissues of angiosperms, with the overwhelming majority of species surveyed to date producing DT (orthodox) seed embryos and pollen [2]. Vegetative DT is a polyphyletic trait in the angiosperms, having evolved independently at least thirteen times [2], with some species retaining chlorophyll (homoiochlorophyllous), whilst other species break chlorophyll down (poikilochlorophyllous) during dehydration. It has been argued that vegetative DT in resurrection plants arose through co-option of the genetic network responsible for the acquisition of DT in orthodox seeds [3]–[5], however the mechanism by which this might have occurred is unknown.

In orthodox seeds, embryonic DT is acquired late in the development process, during seed maturation. Maturation occurs subsequent to embryo and endosperm morphogenesis, and is characterised by embryo growth arrest, reserve accumulation and the acquisition of stress tolerance [6]. In many angiosperm species seed maturation is not required for the generation of a viable embryo: excised, un-matured seeds will still germinate under favourable conditions [7], [8]. Rather, the process of maturation prepares the seed for survival in unfavourable conditions outside of the parent plant by inducing a stress-tolerant, quiescent state.

Embryonic DT occurs as a pre-programmed event rather than as a physiological response to water loss [9], [10]. The exact mechanisms by which orthodox seeds become DT are not yet fully understood, however a number of factors have been shown to correlate strongly with the ability of a seed to survive desiccation. These include the up-regulation of LEA (late embryogenesis abundant) proteins, and the accumulation of sucrose and several oligosaccharides. The appearance of LEA proteins in maturing seeds coincides with the acquisition of DT, and their levels fall once germination has been initiated and tolerance is lost [11]. LEA proteins are hypothesised to function, at least in part, as a “water replacement” mechanism: as water is lost from the cell, the hydrophobic LEAs form a protective layer around exposed chemical groups, preventing potential damage or loss of conformation [11]–[13]. Carbohydrates - primarily sucrose, but also oligosaccharides such as raffinose and stachyose - show a marked increase in concentration in embryonic tissues during maturation [14]. In addition to acting as a readily-available energy source for the embryo during germination, these sugars are thought to function in a similar fashion to LEAs by acting as water replacement molecules [12], [15].

Mature orthodox seeds can remain quiescent and DT for extremely long periods of time until growth is re-initiated by imbibition. Germination is induced in non-dormant imbibed seeds by gibberellin-regulated gene networks which reduce ABA concentrations in the seed and silence maturation-specific genes, thereby reversing the maturation-induced embryo quiescence [16]. DT is progressively lost during the germination of orthodox seeds, until the seedlings reach a so-called “point of no return” past which they can no longer survive dehydration [17]. For many species, including *Arabidopsis thaliana*, this point is reached early during the imbibition stage prior to visible protrusion of the radicle through the testa [18], [19]. In other species, however, the point of no return can be extended until well after radicle emergence. This phenomenon is often seen in desert plant species, where the ability for a seedling to survive sudden and unexpected drought conditions unharmed would confer a significant selective advantage [20]–[22].

Exposure to moderate osmotic stress, such as incubation in high molecular weight polyethylene glycol (PEG), can extend the developmental window during which DT can be re-established in germinating seedlings of many species [23], albeit narrowly in many cases. For example, in *Medicago truncatula* DT is rapidly lost following germination; only 20% of seedlings with radicles >2 mm and 0% with >3 mm in length were able to re-establish DT [24]. However, following pre-treatment with -1.7 MPa PEG these values increased to 100% and 72% respectively [24]. Notably, the survival rate of seedlings with radicles >4 mm in length remained at 0%. Similarly, in *Tabebuia impetiginosa,* untreated seedlings are unable to re-establish DT once radicle length reaches 2.5 mm, while >50% of seedlings pre-treated with -1.7 MPa PEG can do so at this developmental stage [25]. In *A. thaliana*, while loss of DT occurred at the time of testa rupture in untreated seedlings, pre-treatment with -2.5 MPa PEG allowed re-establishment of DT in >95% of seedlings with radicles 0.3–0.5 mm in length, and in 38% of seedlings displaying root hair formation [18].

Transcriptome analysis of *M. trunculata* seedlings undergoing re-establishment of DT following PEG treatment revealed a significant overlap with changes in gene expression observed during the establishment of DT during seed maturation [26]. Similarly, analysis of gene expression in *A. thaliana* seedlings revealed that genes encoding LEA, seed storage and dormancy related proteins were strongly up-regulated following PEG treatment, while those involved in energy metabolism and cell-wall modification were repressed [18]. Together these data suggest that re-establishment of DT in seedlings involves a return to a quiescent state similar to that occurring in mature orthodox seeds.

If vegetative DT has indeed evolved in resurrection plants via co-option of the genetic network responsible for the acquisition of DT in seeds, one possible mechanism by which this may have occurred is through extension of the developmental window during which seedling re-establishment of DT can occur. If this is so, we would predict that the seedlings of resurrection plants would not display a point of no return, and would instead be able to re-establish DT throughout germination and beyond. In order to test this prediction, we characterised the germination process of seeds of the resurrection plant *Xerophyta viscosa* (Velloziaceae), and compared the ability of seedlings of *X. viscosa* and the model plant *A. thaliana* to re-establish DT following germination.

## Materials and Methods

### Plant material

*X. viscosa* (Baker) seeds were obtained from Silverhill seeds (http://www.silverhillseeds.co.za/default.asp), from a batch of seeds collected from Witsieshoek (Free State province, South Africa). Seeds were stored in the dark at room temperature. *Arabidopsis thaliana* seeds of the Columbia (Col-0) ecotype were obtained from the European Arabidopsis Stock Centre (http://arabidopsis.info) and stored at 4°C in the dark.

### Germination and growth conditions

Seeds of both species were surface sterilised with 75% (v/v) ethanol for 2 min and air-dried in a laminar flow hood. Seeds were germinated on half-strength Murashige and Skoog (MS) medium containing 0.7% (w/v) bacteriological agar. Unless specified otherwise, seeds of *X. viscosa* were germinated immediately while *A. thaliana* seeds were cold-stratified at 4°C for 48 h in the dark prior to germination. Plants were grown under a long-day photoperiod (16 h light, 8 h dark) at 22°C, and cool white fluorescent light of 250 μmol m^−2^s^−1^.

### Photography and seedling measurements

Seed and seedling photographs were obtained using a digital colour camera (JVC) mounted on a dissecting stereomicroscope (Olympus). Seedlings were photographed on a single piece of damp filter paper in order to prevent premature dehydration of their tissues due to strong lighting. Cotyledon, root and seed measurements were determined for each seedling using the ImageJ imaging software (http://rsbweb.nih.gov/ij/). High resolution photographs of seeds and seedlings were obtained using a Nikon Stereoscope Zoom Microscope (SMZ1500) and NIS-Elements (Nikon) digital 3D imaging software.

### Response of *X. viscosa* seeds to various dormancy-breaking treatments

Batches of 25-30 seeds were treated with one of a number of dormancy breaking treatments previously described for various plant species [27]: 4°C or 37°C incubation in the dark for 72 h on half strength MS medium plates; 72 h soak in the dark at 4°C or 37°C in either a 1% (w/v) thio-urea solution or 0.25% (w/v) potassium nitrate solution; 72 h stored at −20°C; acid scarification with a 50% (v/v) H_2_SO_4_ solution for 1 minute; 60°C soak in water for either 1 min or 1 h; twelve weeks 4°C moist chilling in constant dark on half-strength MS plates. The treated seeds were subsequently transferred to half-strength MS medium plates (if not already on such a plate) and germinated under standard growth conditions.

### The effect of developmental stage on seedling survival after rapid dehydration

*A. thaliana* seedlings were developmentally staged according to the system previously described by Maia *et al*. [18]. The stages of *X. viscosa* germination have not previously been described, and so this developmental process is described in the results section. For the dehydration experiments, germinated seeds at specific developmental stage were transferred to damp filter paper in an open petri dish and dehydrated under constant air flow in a laminar flow hood. After 48 h, seedlings were rehydrated on the filter paper with 2.5 ml sterile water and the plates transferred back to standard growth conditions. Seedlings were tracked over a 5 d period by daily inspection under an Olympus dissecting light stereo microscope, and considered DT if they resumed normal development.

### The impact of PEG or sucrose treatment on seedling revival after dehydration

For the PEG-treatment, germinated seeds at appropriate developmental stages were moved to filter paper saturated with 1.2 ml of a PEG-8000 solution calculated to have an osmotic potential of -2.5 MPa at 22°C [28], on which they were incubated under standard growth conditions for 48 h. Following PEG incubation, the seedlings were rinsed in sterile water to remove excess PEG and transferred to fresh damp filter paper. Dehydration and rehydration proceeded as described above. For the sucrose treatment experiments, seeds were germinated on half-strength MS in the presence or absence of 3% (w/v) sucrose under standard growth conditions. Germinated seeds at each developmental stage were then transferred to damp filter paper and subjected to dehydration as described above.

### Data analysis

Due to the asynchronous and variable nature of *X. viscosa* germination, coupled with the difficulty in acquiring the seeds of this plant, the experiments described here occurred over the course of multiple weeks in a small number of large-scale experiments involving several hundred seeds. Data from multiple independent experiments was pooled and the variation within the entire pool of samples was determined via bootstrapping. The seedlings in each pooled experiment were resampled with replacement and placed into bins of pre-defined cotyledon lengths. This was repeated 10000 times, and the mean survival rate for the resampled seeds in each bin was calculated for each iteration. The standard error of the mean (SEM) of the sample pools was calculated from the standard deviation of the bootstrapped mean survival values. The same procedure was applied to the data generated for the *Arabidopsis* germination experiments to facilitate comparison.

## Results

### Germination and growth of *X. viscosa* seedlings

**Figure 1:**
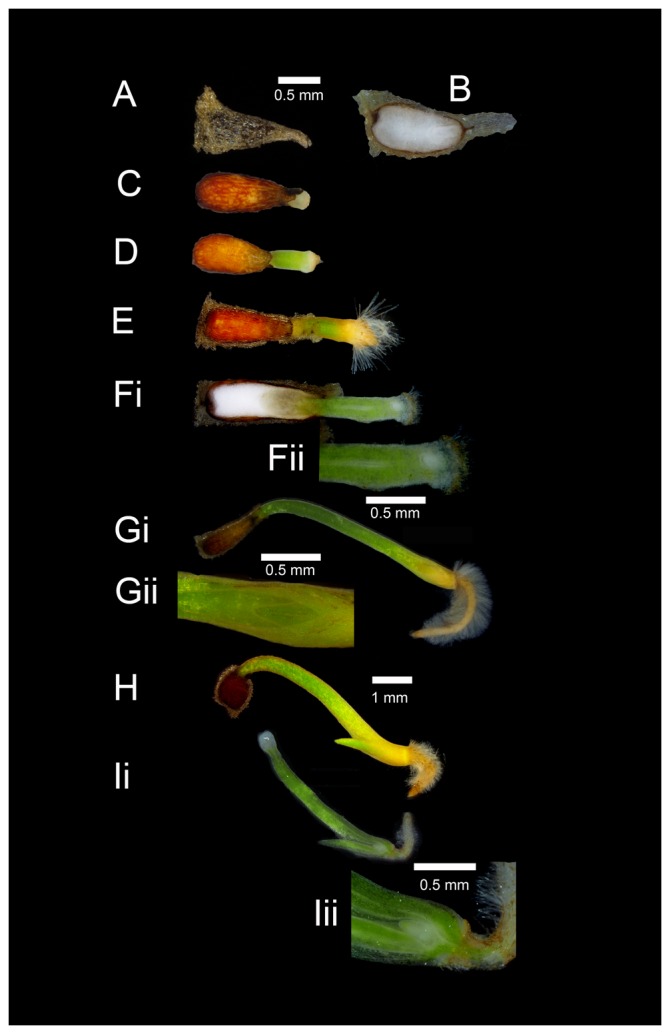
The germination process in *X. viscosa*. (A) dry seed; (B) cross-section of an imbibed seed; (C) radicle emergence; (D) chlorophyll is present in the emerging cotyledon; (E) root hairs appear once the cotyledon is 1 to 3 mm in length; (Fi) cross section of seedling showing tip of the photosynthetic cotyledon in contact with the endosperm, and the pale meristem; (Fii) a central protoxylem connecting the meristem to the tip of the cotyledon is visible; (Gi) the cotyledon and root tip elongate rapidly, and a slit appears above the root through which the first true leaf emerges (Gii); (H) the emerging leaf is clearly visible and the cotyledon ceases to grow. A cross section at this stage (Ii and Iii) shows that the meristem is no longer connected to the endosperm via the protoxylem. Seedlings at this stage are no longer dependent on the endosperm for survival. The 0.5 mm scale bar at the top of the figure is applicable to images A to Fi, and the 1 mm scale bar to images Gi, H and Ii. Individual scale bars are provided for Fii, Gii and Iii.

The germination and subsequent development of *X. viscosa* seedlings has not been previously been reported. We attempted to optimize germination conditions and developed a staging system to describe the growth of seedlings to aid further study on these plants. The seeds of *X. viscosa* are generally elliptical in shape, and surrounded by a triangular, dry, transparent husk that extends from one side of the seed (Figure 1A). When imbibed the seed swells with absorbed water and the embryo, located opposite the origin of the husk, is clearly visible (Figure 1B). The vast majority of the seed volume consists of nutrient endosperm, in which the embryo is embedded, and is surrounded by a thin, brown seed coat. Germination occurs over a period of 4 to 14 days thereafter. Germination rate and synchronicity were only marginally improved by various common dormancy breaking treatments, such as moist chilling (Figure S1). The germination of *X. viscosa* seedlings was found to visibly resemble that of the onion, *Allium cepa*, albeit on a much smaller scale [29]. Initially, the cotyledon lengthens within the seed and forces the developing radicle through the testa (Figure 1C). The embryo is reliant on nutrients from the endosperm for the first few days of germination and embryos excised during this stage are not viable. As germination progresses, the cotyledon continues to lengthen and rapidly turns green (Figure 1D). The radicle develops into a single primary root which develops root hairs once the cotyledons are between 1–3 mm in length (Figure 1E). At this stage, the embryo can be divided into four different zones: the cotyledon tip (a pale translucent ball of tissue that absorbs food stores from the endosperm), photosynthetic cotyledon tissue, the meristem above the roots, and a pointed root tip (Figure 1F). A central protoxylem is visible in cross-section of the germinating seedling, connecting the meristem, above the root zone to the endosperm (Figure 1F). The cotyledon undergoes a period of rapid extension (to 4–5 mm in length), before a split in the leading end of the cotyledon (Figure 1G) heralds the appearance of the first true leaf (Figure 1H). Undifferentiated, pale meristem tissue visible in cross-sections of seedlings above the root is no longer connected to the endosperm via the protoxylem (Figure 1I).

### The effect of developmental stage on seedling survival after rapid dehydration

**Figure 2:**
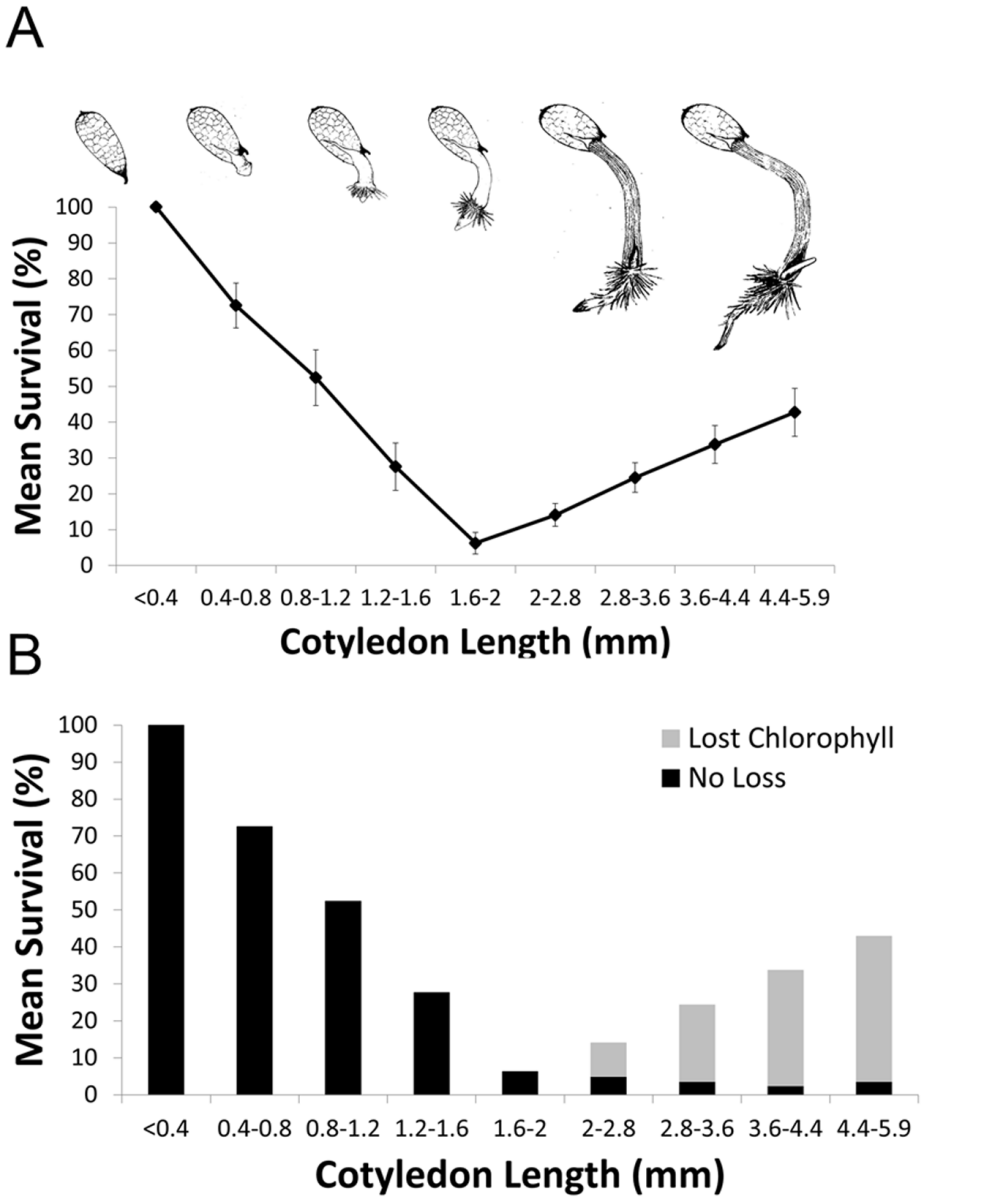
Desiccation tolerance in germinating seedlings of *X. viscosa* and *A. thaliana*. (A) Survival of germinating *X. viscosa* seedlings after 48 h desiccation, grouped by cotyledon length. Data shows the mean survival rate ±SEM as determined by bootstrapping. Representative line drawings of germinating seedlings are shown above. (B) Incidence of chlorophyll degradation in the surviving seedlings from Figure 2A. Black bars: survived and did not lose chlorophyll. Grey bars: survived and lost chlorophyll. The numbers of seeds per bin are provided in Table S1.

Mature leaves of adult *X. viscosa* are desiccation tolerant [30]. We set out to determine whether germinating seedlings of *X. viscosa* are initially DT and whether this property was lost as seedlings matured through to the appearance of the first true leaves. We found that *X. viscosa* seedling survival was strongly associated with the seedling size at the time of drying (Figure 2A). Seedlings at an early stage of germination (i.e. cotyledon <0.4 mm) showed a near-100% survival rate. Survival rate decreased as seedlings increased in size, dropping to only 6% in seedlings with cotyledons 1.6–2 mm in length. Survival rate began to increase again, however, in larger seedlings (cotyledons >2 mm), rising to over 40% survival in the oldest tested seedlings (cotyledon >4.4 mm, around the stage at which the primary leaf emerges).

**Figure 3:**
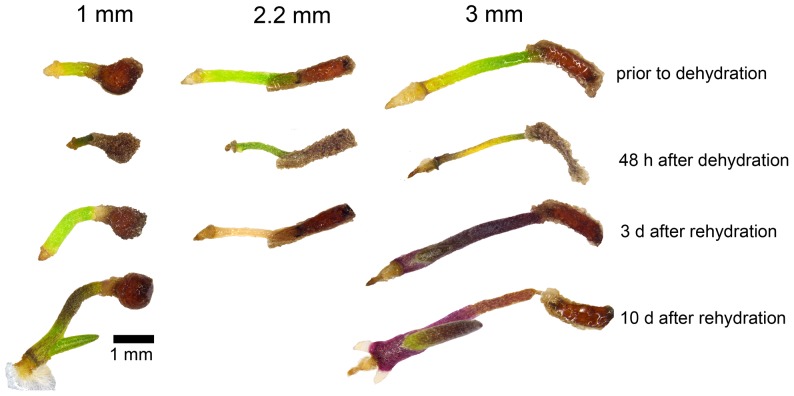
Recovery of *X. viscosa* seedlings from dehydration. Representative images of three untreated *X. viscosa* seedlings 1 mm, 2.2 mm and 3 mm in length before and after dehydration and rehydration. The 1 mm seedling dehydrated without chlorophyll loss, but recovered and resumed growth rapidly after rehydration. A 2.2 mm seedling likewise failed to degrade chlorophyll, but did not survive the rehydration process. A 3 mm seedling displayed partial chlorophyll loss from its cotyledon during dehydration. The seedling survived with its meristem, primary leaf and leading end of the cotyledon intact; the primary root failed to survive, but two additional secondary roots emerged from the meristem. Anthocyanins accumulated in the photosynthetic tissue during rehydration of this seedling.

**Figure 4:**
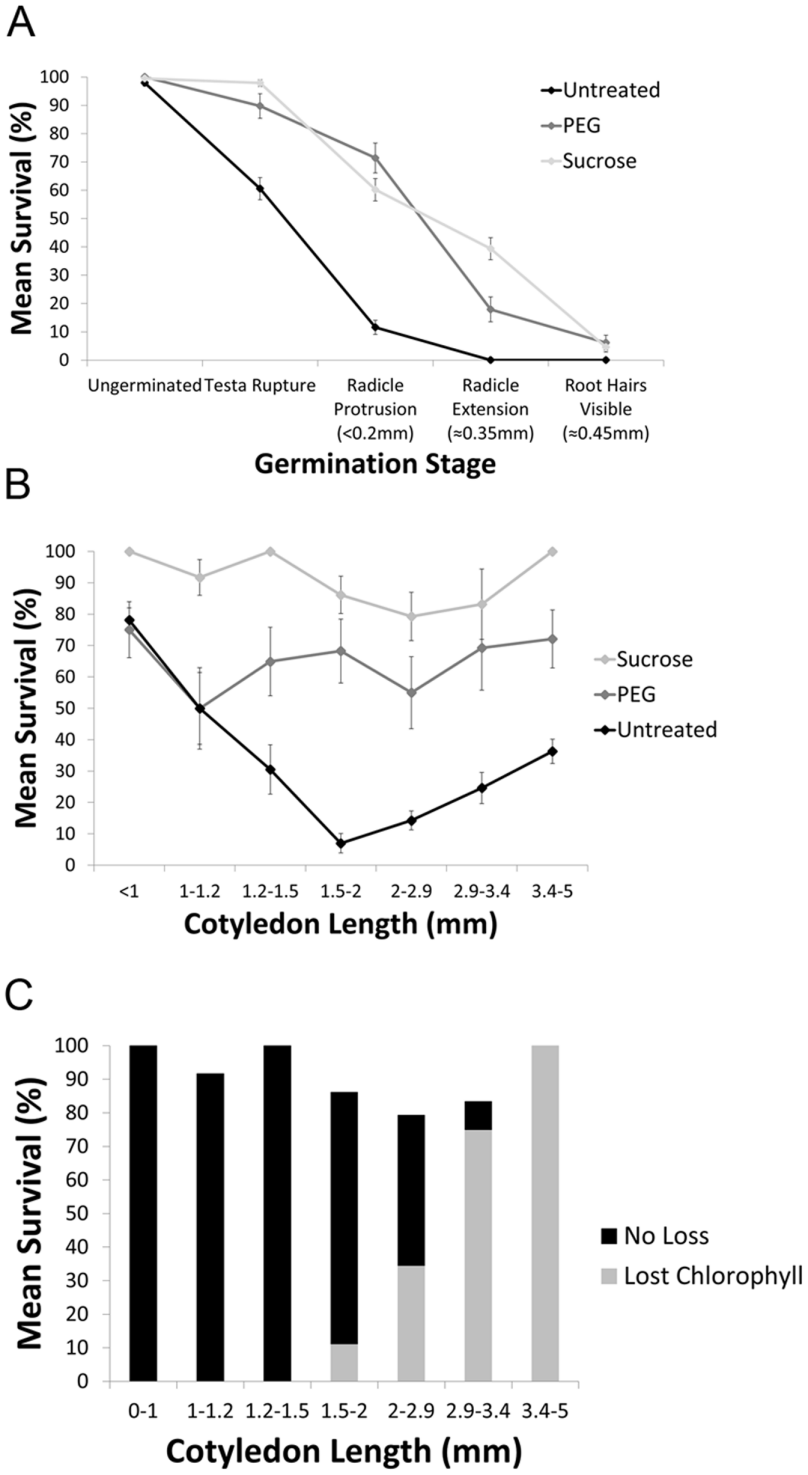
Pre-treatment with PEG or sucrose improves desiccation tolerance in germinating seedlings of *A. thaliana* and *X. viscosa*. (A) Mean survival rate (±SEM) of *A. thaliana* seedlings after 48 h desiccation at various stages of germination. Although survival improves with PEG and sucrose treatment, *A. thaliana* seedlings are unable to survive desiccation past the “root hairs visible” seedling stage. (B) Survival of germinating *X. viscosa* seedlings with or without PEG or sucrose pre-treatment, grouped by cotyledon size (±SEM). Seedlings pre-treated with either sucrose or PEG did not show a decline in desiccation tolerance compared to untreated seedlings. (C) Chlorophyll loss in *X. viscosa* sucrose-treated seedlings. Although the mean survival rate of sucrose-treated seedlings was relatively consistent across most stages, the incidence of chlorophyll loss increased steadily with increasing seedling size. Seeds per bin can be found in Table S1.

Small seedlings (cotyledons <2 mm) displayed no visible predictors of survival: the cotyledons of desiccated seedlings remained green, and cotyledon and root tissues withered rapidly from lack of water (Figure 2B & 3). In contrast, survival of seedlings with cotyledons >2 mm was associated with the loss of chlorophyll either wholly or partially from the cotyledon (Figure 2B & 3). Of seedlings with cotyledons >2 mm, a total of 30% lost chlorophyll of which 76% survived dehydration, whereas only 5% of seedlings that did not lose chlorophyll at these stages survived. In a number of cases, tissue survival was restricted to regions that had degraded chlorophyll, while surrounding tissues failed to revive (Figure S2). The primary root of surviving seedlings usually also survived the desiccation process unharmed; in cases where it did not, a secondary root would erupt next to or through the dead root tissue (Figure 3). As has been previously reported [18], we observed that *A. thaliana* seedlings lost DT rapidly once the seed coat had ruptured. Less than 10% of seedlings survived 48 h of dehydration once the radicle had visibly protruded through the seed coat, with the survival rate dropping to 0% at all subsequent developmental stages (Figure 4A).

### The impact of PEG or sucrose pre-treatment on seedling survival after dehydration

The transient decline in the ability of *X. viscosa* seedlings to survive dehydration during early germination (Figure 2A) might represent a developmental stage at which DT generally cannot be re-established. Alternatively, these young seedlings may be particularly vulnerable as they do not have sufficient resources to mount a successful response against or recover from very rapid dehydration. For example, depletion of the endosperm-derived nutrients upon which the germinating seedlings are dependent during initial growth, or a failure to protect the protoxylem connecting the endosperm to the meristem, may be lethal. The point of no return can be delayed in many species by priming germinating seedlings with moderate osmotic stress prior to dehydration, for example by incubating in high molecular weight PEG. Sucrose has also been implicated in the survival of both germinating seedlings and resurrection plants in response to desiccation, and the presence of additional sucrose may alleviate resource depletion in germinating seedlings that have not yet become self-sufficient. We thus used PEG and sucrose pre-treatment to investigate whether priming of *X. viscosa* or *A. thaliana* seedlings improved their ability to survive dehydration.

In agreement with previous reports, incubation in a -2.5MPa PEG-8000 solution for 48 h prior to dehydration improved the survival rate of *A. thaliana* seedlings (Figure 4A), most noticeably at the stages of radicle protrusion (radicle 0.2 mm; 9% survived untreated vs. 74% PEG-treated) and radicle extension (radicle 0.35 mm; 1% vs. 20%). Nonetheless, mean survival rate dropped to 5% by the time the root hairs were first visible (radicle 0.45 mm). Growth on 3% sucrose resulted in delayed germination in *A. thaliana* and led to the accumulation of anthocyanins, as previously reported [31], [32]. However, the presence of exogenous sucrose also improved seedling survival after dehydration. Whereas only 9% and 1% of untreated seedlings survived at the stages of radicle protrusion and extension respectively, supplementary sucrose improved survival to 59% and 42% at these stages (Figure 4A). Mean survival dropped to less than 5% in seedlings with a radicle longer than 0.45 mm.

**Figure 5:**
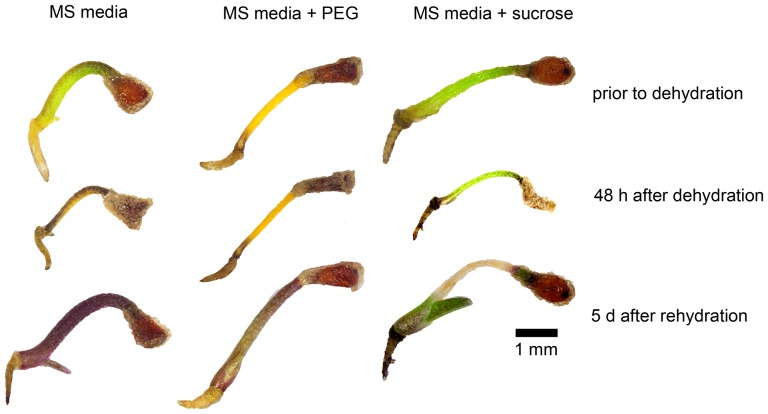
Pre-treatment of *X. viscosa* seedlings with PEG results in chlorophyll degradation. Representative images of an untreated, PEG-treated and sucrose-treated seedling undergoing dehydration and rehydration. An untreated seedling (MS media) lost chlorophyll and accumulated anthocyanins in response to dehydration, and survived rehydration. A PEG treated seedling completely degraded its chlorophyll in response to PEG priming (prior to dehydration), and recovered well after rehydration. A sucrose-treated seedling failed to lose chlorophyll during dehydration; however, the meristem, primary leaf and a small portion of the cotyledon recovered after rehydration nonetheless.

PEG treatment also increased seedling survival rate after dehydration in *X. viscosa* (Figure 4B). Total seedling survival averaged 65%, and the transient decline in survival rate observed in untreated plants was not apparent in PEG-treated seedlings. Notably, the majority (83%) of seedlings of all sizes showed complete loss of chlorophyll after incubation in PEG (Figure 5). None of the 17% of PEG-treated seedlings that failed to degrade chlorophyll were able to survive dehydration. In contrast to its effects on delaying germination in *A. thaliana*, 3% sucrose did not have any noticeable effect on *X. viscosa* germination rate, nor did it induce anthocyanin accumulation in seedlings prior to dehydration (Figure 5). However, it drastically improved seedling survival irrespective of cotyledon length: average survival of sucrose-treated seedlings was 91% across all developmental stages, with the lowest survival rate of 79% occurring in seedlings 2–2.9 mm in size (Figure 4B). As with untreated plants, chlorophyll loss was only observed in seedlings with larger cotyledons (>1.5 mm), and occurred most often (>90%) in seedlings >3 mm in length (Figure 4C).

## Discussion

The seeds of the desiccation-sensitive plant *A. thaliana* can halt the process of germination under adverse conditions, with a window of opportunity of 48–60 h post imbibition [33]. Current evidence suggests that this process involves resetting the seedling to a quiescent state that resembles that of mature, dry orthodox seeds [18]. A similar window is seen in many other angiosperm plant species. The acquisition of vegetative DT in angiosperms may have evolved through the co-option of the networks regulating angiosperm seed maturation genes into adult tissues [2], [4]. A possible means by which this could have occurred would be through extending the temporary window during which angiosperm seedlings are able to re-establish DT indefinitely. If this is the case, we would predict that the seedlings of a resurrection plant would not display a point of no return, but would instead be desiccation tolerant throughout germination. Our aim was to determine the extent to which this window of opportunity existed in the monocot resurrection plant *X. viscosa*, a poikilochlorophyllous resurrection plant from Southern Africa. We investigated the DT of the seedlings of both *X. viscosa* and the desiccation sensitive *A. thaliana*, when untreated or either incubated in PEG or supplemented with exogenous sucrose.

In agreement with previous reports, we found that the seedlings of *A. thaliana* rapidly lost DT once the seed coat had ruptured (Figure 4A), but could be temporarily rescued by pre-treatment in a high molecular weight PEG solution prior to desiccation [18]. Growth on medium supplemented with 3% sucrose, though it delayed germination and retarded seedling development, was also capable of increasing survival rates in germinating seedlings to a similar degree. However, neither PEG nor sucrose treatment could substantially extend the window beyond the point at which radicle length >0.45 mm (Figure 4A).

*X. viscosa* seedlings grown on half-strength MS media displayed a transient decline in DT during germination. Although freshly imbibed seedlings were desiccation tolerant, seedlings steadily lost DT as germination progressed, dropping to a minimum survival rate of 6% in seedlings between 1.6–2 mm in length (Figure 2A). However, this was a temporary phenomenon, and a greater proportion of seedlings recovered from dehydration at later stages, rising to over 40% survival in seedlings >4.4 mm. The majority of surviving seedlings greater than 2 mm in length also lost chlorophyll from their photosynthetic tissues (Figure 2B), a trait reminiscent of the mechanism of poikilochlorophylly employed by mature *X. viscosa* plants to prevent build-up of reactive oxygen species during dehydration [34].

Pre-treatment of germinating *X. viscosa* seedlings with PEG for 48 h prior to desiccation reduced the transient decline in DT (Figure 4B), and improved the average survival rate over that of untreated seedlings by a large margin (minimum survival rate was over 50%, compared to 6% in untreated seedlings). Interestingly, all surviving seedlings showed a complete loss of chlorophyll from the PEG treatment alone, irrespective of seedling size. Sucrose-supplemented *X. viscosa* seedlings displayed an even more marked increase in survival: on average 91% of total seedlings recovered completely after desiccation, with a minimum survival rate of 79% in seedlings 2–2.9 mm in length (Figure 4B). As with untreated seedlings, only sucrose-treated seedlings with a cotyledon >2 mm displayed any degradation of chlorophyll during dehydration, with the incidence of chlorophyll loss increasing as seedlings increased in size (Figure 4C).

Moderate osmotic stress during the early stages of germination has been shown to improve the survival of desiccated seedlings in a number angiosperm species [23], [24]. The exact mechanism through which this occurs is not clear, but it seems likely that an extended period of moderate stress primes the seedlings to activate the seed maturation genes and desiccation response mechanisms prior to the period of rapid desiccation. On the other hand, treatment with exogenous sucrose has to our knowledge not been previously shown to induce DT in germinating seedlings, although it has been reported to induce DT in axillary buds and shoot tips of various species for the purposes of cryopreservation [35], [36].

As PEG treatment alone was sufficient to substantially rescue *X. viscosa* seedlings at the stages at which they were most sensitive to dehydration, it is unlikely that the transient loss of DT in untreated seedlings was due to an inherent inability of these seedlings to induce DT. Some other factor, such as depletion of endosperm-derived nutrients, or increased dehydration rate due to a larger surface-area-to-volume ratio, may have played a role in reducing the survival rate of seedlings at these sizes. Whilst it is well known that lower plants, such as the moss *Tortula ruralis*, can withstand very rapid water loss, this is not possible for angiosperm resurrection plants which need time to lay down protective measures [37]. We hypothesise that pre-treatment with PEG primed the desiccation response prior to the rapid dehydration treatment. Consistent with this hypothesis, all surviving PEG-treated seedlings showed a loss of chlorophyll from the cotyledon, indicating that *X. viscosa* is capable of poikilochlorophylly at all stages of germination. This suggests either a direct relationship between the *X. viscosa* seedling and vegetative desiccation response mechanisms, or alternatively that the adult response mechanisms can be induced in seedlings under the appropriate conditions. None of the PEG-treated seedlings that failed to lose chlorophyll (17% of the total) survived the dehydration treatment, strong evidence that chlorophyll degradation is an indicator of the preparedness of a seedling to respond to rapid, severe water loss.

In contrast, small (<2 mm) untreated and sucrose-treated seedlings failed to degrade chlorophyll during dehydration. This observation may be explained by the rate of water loss in these seedlings being too rapid for this process to occur without PEG priming due to the seedlings' small size. However, the survival of these small seedlings without chlorophyll loss suggests that the mechanisms that control vegetative DT and poikilochlorophylly do not occur simultaneously; rather DT protection is activated prior to visible loss of chlorophyll from these tissues. Presumably, in non-PEG-treated seedlings >2 mm in length, their increased size sufficiently delayed the rate of dehydration for chlorophyll degradation to begin to occur in these tissues in the absence of any priming.

Sucrose treated seedlings showed an even greater survival rate than that of PEG treated seedlings in both species, despite the fact that they should have had a similar dehydration rate to untreated seedlings. Thus, the rate at which seedlings lose water is not the only factor influencing the probability of recovery. The exact role exogenous sucrose plays in this process cannot be determined from the results in this study. It seems unlikely that the relatively minor osmotic effects of sucrose alone (−0.22 MPa) [38], would be sufficient to prime the DT response, as osmotically stressed PEG-treated seedlings (−2.5 MPa) did not recover to a similar degree. Exogenous sucrose may have acted as a nutrient source that was independent of seedling stage, reducing the total number of seedlings that failed to survive purely due to a lack of sufficient resources to mount a successful desiccation response. Sucrose has also been predicted to act as an osmoprotectant that, together with LEA proteins, forms the cellular glass that protects tissues from desiccation [39]. For example, it is possible that the exogenous sucrose protected the protoxylem which serves as a lifeline between the endosperm reserves and the meristem in small seedlings. Alternatively, high background levels of intra- or extracellular sucrose could have also reduced the time needed to induce glass formation during the desiccation process. Treatment with exogenous sucrose has been shown to have a synergistic effect with ABA in inducing DT in seeds or somatic embryos, if applied during the early stages of the maturation process [40]–[42]. If the window of seedling DT does involve an ABA-mediated reactivation of maturation genes, high sucrose levels may have had a similar synergistic signalling role with ABA. None of these roles are mutually exclusive, and it is likely that exogenous sucrose functions through multiple pathways to improve survival rates of desiccating seedlings.

Our data suggest that *X. viscosa* seedlings do not inherently lose DT during germination, as other angiosperm species do. A particularly striking result is the potential for poikilochlorophylly at all seedling stages, suggesting a direct relationship between the seedling and adult desiccation response. One explanation for these results is that *X. viscosa* never closes the window of ABA responsiveness that allows germinating angiosperm seeds to revert to a quiescent, mature-dry state, but has instead evolved the ability to activate this response throughout its life, even in adult tissues. The acquisition of additional molecular data and functional assays will be informative in determining whether the vegetative desiccation response in *Xerophyta* is indeed an extension of the germinating seedling desiccation response, or whether these are different survival programmes.

## Supporting Information

Figure S1 **The effect of various common dormancy-breaking treatments on seeds of** ***X. viscosa*** **.** Data shown are the percentage germination over time from batches of 25 to 50 seedlings per experiment. (A) Dormancy-breaking treatments coupled with cold (4°C or freezing) temperature. (B) Dormancy-breaking treatments coupled with warm (37°C) temperature. (C) Extended 4°C stratification coupled with germination in light or dark conditions. Not shown: acid scarification (50% H_2_SO_4_, 1 minute) and 60°C soak, which resulted in 100% mortality. While the majority of treatments advanced the germination process by 24 h (possibly due to advanced period of imbibition compared to untreated seedlings), neither the rate of germination nor the total percentage of seeds germinating was greatly enhanced.
(TIF)

Figure S2 **Chlorophyll loss is a predictor of tissue survival in older seedlings.** Chlorophyll degradation and subsequent recovery (indicated by black arrows) of seedling tissues was a strong predictor of survival after dehydration. A) A desiccated seedling shows chlorophyll loss around the meristem, a small emerging leaf, and the upper cotyledon. B) These tissues recover 5 days post rehydration, the meristem survives, and the emerging leaf is substantially larger. C) A desiccated seedling showing chlorophyll loss from only the upper cotyledon D) Whilst chlorophyll is restored in this section of the cotyledon, the meristem and lower cotyledon fail to survive.
(TIF)

Table S1 **Seedling bin sizes for** **figures 2A** **,** **4A and 4B** **.** The seedlings from independent experimental repeats were pooled and divided into bins based on cotyledon length (*X. viscosa*) or germination stage (*A. thaliana*).
(XLSX)
