# Supplementary figures and images for: The Window of Desiccation Tolerance Shown by Early-Stage Germinating Seedlings Remains Open in the Resurrection Plant, Xerophyta viscosa
Source: PLoS One. 2014 Mar 25;9(3):e93093. doi: 10.1371/journal.pone.0093093 (PMC3965527; doi:10.1371/journal.pone.0093093)

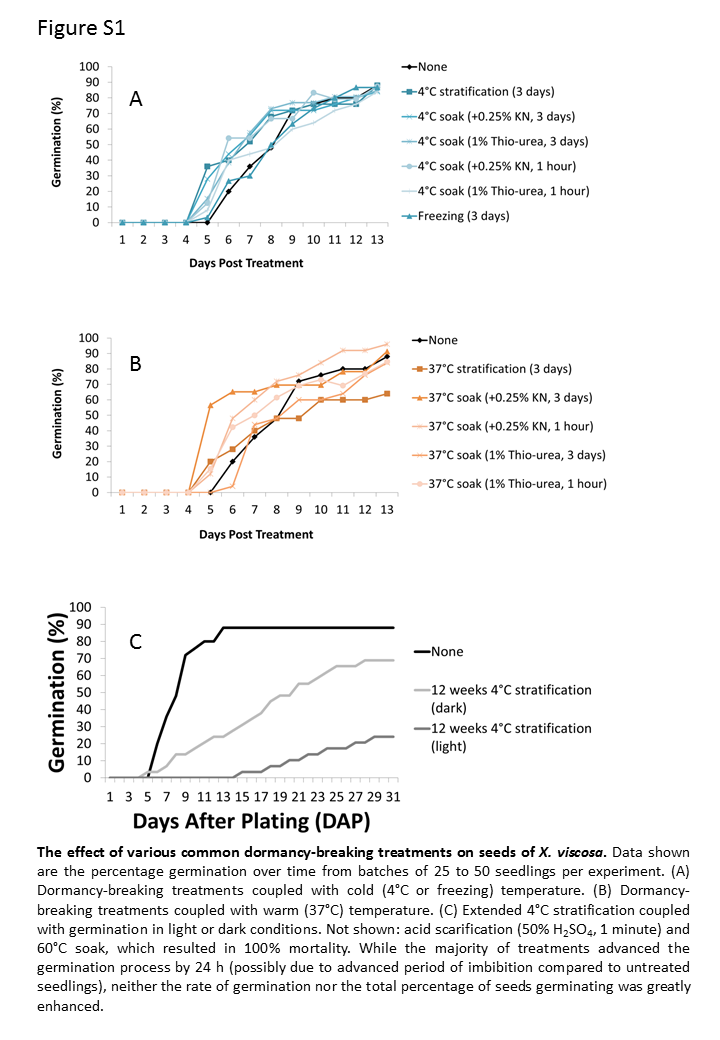

Supplement: Figure S1 — The effect of various common dormancy-breaking treatments on seeds of X. viscosa . Data shown are the percentage germination over time from batches of 25 to 50 seedlings per experiment. (A) Dormancy-breaking treatments coupled with cold (4°C or freezing) temperature. (B) Dormancy-breaking treatments coupled with warm (37°C) temperature. (C) Extended 4°C stratification coupled with germination in light or dark conditions. Not shown: acid scarification (50% H2SO4, 1 minute) and 60°C soak, which resulted in 100% mortality. While the majority of treatments advanced the germination process by 24 h (possibly due to advanced period of imbibition compared to untreated seedlings), neither the rate of germination nor the total percentage of seeds germinating was greatly enhanced. (TIF) [file pone.0093093.s001.tif]

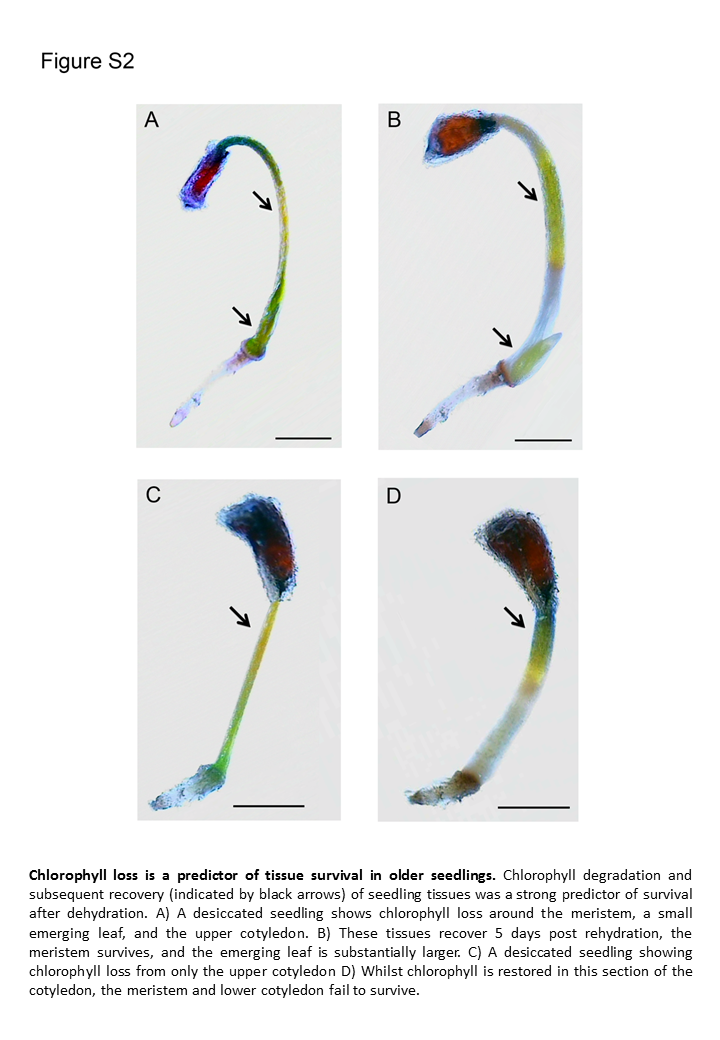

Supplement: Figure S2 — Chlorophyll loss is a predictor of tissue survival in older seedlings. Chlorophyll degradation and subsequent recovery (indicated by black arrows) of seedling tissues was a strong predictor of survival after dehydration. A) A desiccated seedling shows chlorophyll loss around the meristem, a small emerging leaf, and the upper cotyledon. B) These tissues recover 5 days post rehydration, the meristem survives, and the emerging leaf is substantially larger. C) A desiccated seedling showing chlorophyll loss from only the upper cotyledon D) Whilst chlorophyll is restored in this section of the cotyledon, the meristem and lower cotyledon fail to survive. (TIF) [file pone.0093093.s002.tif]
